# Changes in serum symmetric dimethylarginine concentrations after treatment of feline hyperthyroidism with antithyroid medications

**DOI:** 10.1177/1098612X261418859

**Published:** 2026-01-21

**Authors:** Sarah E Cox, Emma L Tarrant, Timothy L Williams

**Affiliations:** 1 Department of Veterinary Medicine, University of Cambridge, Cambridge, UK; 2 The Ralph Veterinary Referral Centre, Fourth Avenue Globe Business Park, Marlow, UK

**Keywords:** Symmetric dimethylarginine, creatinine, chronic kidney disease, hyperthyroidism, methimazole, carbimazole

## Abstract

**Objectives:**

The aim of the present study was to report changes in serum creatinine and symmetric dimethylarginine (SDMA) concentrations after treatment of feline hyperthyroidism with anti thyroid medications and to compare these biomarkers at baseline between cats that were and were not azotaemic after treatment.

**Methods:**

In this retrospective study, hyperthyroid cats that were euthyroid (total thyroxine [TT4] concentration 7–40 nmol/l) at 1 month (T1) and/or 2–9 months (T2) after treatment were identified and grouped by renal status defined by serum creatinine concentrations. Comparisons were made using non-parametric statistics and the correlations assessed using Spearman’s correlation coefficient. Data are presented as median (minimum–maximum).

**Results:**

A total of 19 hyperthyroid cats were included. At baseline, TT4 was negatively correlated with serum concentration of creatinine (*r*_s_ = −0.73; *P* <0.001) but not SDMA (*r*_s_ = −0.42; *P* = 0.074). Serum creatinine concentrations increased significantly at T1 and T2 (137 μmol/l [range 97–241] and 162 μmol/l [range 76–251]) compared with baseline (117 μmol/l [range 62–216]; *P* = 0.003 and *P* <0.001, respectively), whereas serum SDMA did not change significantly at T1 but did increase by T2 (11 μg/dl [range 8–29] and 13 μg/dl [range 9–24], respectively) compared with baseline (12 μg/dl [range 7–21]; *P* = 0.548 and *P* = 0.039, respectively). There was no significant difference in baseline serum SDMA between cats that were azotaemic after treatment and those that remained non-azotaemic (12 μg/dl [range 7–21], n = 13 vs 13 μg/dl [range 11–19], n = 6; *P* = 0.42).

**Conclusions and relevance:**

Serum SDMA concentrations are not helpful in predicting post-treatment azotaemia in initially non-azotaemic hyperthyroid cats treated with antithyroid medications and might be influenced by factors other than glomerular filtration rate in hyperthyroidism.

## Plain language summary

Assessment of serum SDMA concentrations in hyperthyroid cats

This study evaluated whether the concentrations of a biomarker of kidney function called symmetric dimethylarginine (SDMA) in the blood change in hyperthyroid cats after treatment. SDMA concentrations did change after treatment but not as quickly as we would expect, which could suggest that SDMA concentrations are affected by factors other than just kidney function.

## Introduction

Hyperthyroidism is the most common endocrinopathy in geriatric cats, and although there is significant geographical variance, studies assessing populations of older cats typically indicate a prevalence in the order of 10%.^1–5^ Chronic kidney disease (CKD) is also prevalent in older cats and approximately 10% of untreated hyperthyroid cats have azotaemic CKD, while a further 15% of medically treated hyperthyroid cats become azotaemic after treatment of hyperthyroidism (referred to as ‘masked’ CKD).^6^ The hypermetabolic state associated with hyperthyroidism results in an increased renal glomerular filtration rate (GFR) and reduced muscle mass, thus decreasing serum creatinine and urea concentrations.^7–12^ These changes therefore complicate the diagnosis of CKD in hyperthyroid cats before treatment. It is established that the presence of pretreatment azotaemia negatively impacts the survival of hyperthyroid cats;^6^ however, the development of azotaemia after treatment in previously non-azotaemic cats is not associated with reduced survival times.^13^ Nevertheless, identifying cats at a higher risk of developing azotaemia after treatment could inform management decisions and enable earlier dietary modification, which can improve long-term survival.^14^

Symmetric dimethylarginine (SDMA) is a by-product of L-arginine methylation that is released into the circulation by all nucleated cells after proteolysis. SDMA is excreted predominantly by the kidneys and, as a result, serum SDMA concentrations are inversely correlated with GFR in healthy cats.^15,16^ Serum SDMA concentrations are also not associated with changes in muscle mass in cats.^17^ Serum creatinine and SDMA concentrations increase after the restoration of euthyroidism in radioiodine-treated hyperthyroid cats;^18–20^ however, in some cats, serum SDMA concentrations decrease after radioiodine treatment.^20,21^ In addition, a poor correlation between serum SDMA concentrations and GFR has been demonstrated in hyperthyroid cats, which suggests that factors other than GFR could affect SDMA concentrations.^21,22^ Altered protein metabolism associated with the hyperthyroid state could result in increased SDMA production and release, or hyperthyroidism could be associated with decreased extra-renal degradation and clearance of SDMA.

The effects of radioiodine treatment and bilateral thyroidectomy on serum SDMA concentrations have been reported previously;^18–24^ however, the effects of methimazole and carbimazole treatment of hyperthyroid cats on serum SDMA concentrations have not yet been characterised. Therefore, investigation of the change in serum SDMA concentrations after treatment of hyperthyroidism with antithyroid medications would be of interest to document whether these differ from those of cats treated with radioiodine.

The aim of this study was to investigate the change in serum creatinine and SDMA concentrations in a population of client-owned hyperthyroid cats treated with methimazole or carbimazole. We hypothesised that serum SDMA concentrations would increase in parallel with serum creatinine after the restoration of euthyroidism due to the reduction in GFR. We also hypothesised that baseline serum SDMA concentrations would be higher in cats with concurrent or ‘masked’ CKD.

## Materials and methods

### Case selection

Ethical approval was granted by the Ethics and Welfare Committee of the Department of Veterinary Medicine, University of Cambridge. The university teaching hospital clinical pathology database was searched for feline serum samples with a total thyroxine (TT4) concentration above the laboratory reference interval (RI) derived in a population of healthy older cats (7–45 nmol/l)^25^ in the 10-year period between 2014 and 2024. These cases were screened for inclusion based on signalment, age, clinical signs, medications, laboratory testing data, comorbidities and type of treatment. Laboratory testing data were extracted from records from before and after treatment, including serum concentrations of TT4, creatinine, urea and, where available, urine specific gravity (USG).

For inclusion in the study, cats were required to have clinical signs and/or physical examination findings that were consistent with hyperthyroidism, a TT4 above 45 nmol/l (upper limit of laboratory RI) and to have subsequently started medical treatment with oral carbimazole or methimazole. Euthyroidism (defined as serum TT4 7–40 nmol/l) must have been demonstrated at 1 month (T1) and/or at 2–9 months (T2) after initiating treatment. Cats were excluded if they were deemed to be systemically unwell, if follow-up blood work at these time points demonstrated inadequate control of hyperthyroidism or if there was insufficient residual stored serum for SDMA analysis. Azotaemia was defined as a serum creatinine concentration above the laboratory RI of 56–153 µmol/l).

### Clinicopathological assays

Blood samples were centrifuged within 2 h of collection and residual serum frozen at −80°C within 24 h of initial biochemical analysis. In most instances, serum TT4 and creatinine were measured contemporaneously using a previously validated human TT4 enzyme immunoassay^25^ and a commercially available spectrophotometric assay for creatinine (OSR6178; Beckman Coulter). Serum SDMA concentrations were measured on residual frozen serum samples up to 10 years after collection using a competitive homogenous immunoassay (IDEXX Laboratories).^26^

### Classification of hyperthyroid cats

Hyperthyroid cats were divided into three groups based on serum creatinine concentration before and after treatment. Non-azotaemic hyperthyroid cats had a serum creatinine below 153 µmol/l (upper limit of laboratory RI) both before treatment and at the T2 (2–9 months) post-treatment time point. Masked azotaemic hyperthyroid cats had a serum creatinine below 153 µmol/l at baseline but had a serum creatinine above 153 µmol/l at T1 and/or T2. Azotaemic hyperthyroid cats had a serum creatinine above 153 µmol/l at baseline and at T1 and/or T2.

### Statistical analysis

Statistical analysis was performed using commercially available software (SPSS Statistics for Windows v27.0; IBM). Results are reported as median (minimum–maximum) unless otherwise specified. The data were assessed for normality by visual histogram assessment and Shapiro–Wilk testing; the data were not normally distributed and non-parametric tests were used. Baseline continuous variables (serum creatinine, urea, TT4 and SDMA concentrations) were compared between groups using the Mann–Whitney U-test or Kruskal–Wallis test. Comparisons within groups at different time points were analysed using the Wilcoxon signed rank test. Correlations between SDMA, TT4 and creatinine were assessed using Spearman’s rank correlation coefficient. Statistical significance was defined as *P* <0.05.

## Results

A total of 19 cats were included in the study (see S1 in the supplementary material). Of them, 14 (74%) were female spayed cats and five (26%) were male castrated cats. All cats were domestic shorthairs or longhairs, and the average age was 14 years (range 8–19). Only five (26%) cats had a recorded USG before initiation of treatment of hyperthyroidism. Comorbidities at the time of diagnosis of hyperthyroidism included hepatic disease (n = 4), gingivitis (n = 3), steroid-responsive skin disease (n = 1), controlled hypertension (n = 1), hypertrophic cardiomyopathy (diagnosed or suspected, n = 3) and chronic diarrhoea (n = 1). Medications at the time of baseline sampling included amlodipine (n = 1, Amodip; Ceva Animal Health), benazepril (n = 1, Fortekor; Elanco UK AH), furosemide (n = 2, generic brands), clindamycin (n = 1, Antirobe; Zoetis) and meloxicam (n = 1, Metacam; Boehringer Ingelheim). In total, 10 hyperthyroid cats were treated with oral methimazole (either Felimazole [Dechra Veterinary Products] or Thyronorm [Norbrook Laboratories]) and nine cats were treated with oral carbimazole (Vidalta; MSD Animal Health UK).

### Baseline biochemical data

Of the 19 hyperthyroid cats in this study, six (32%) were classified as non-azotaemic, nine (47%) were classified as masked azotaemic and four (21%) were classified as azotaemic. Of the masked azotaemic group, three were azotaemic and four were non-azotaemic at T1, and seven were azotaemic at the T2 time point. Two masked azotaemic cats that were azotaemic at T1 had no data at T2 and one of these cats was azotaemic at both T1 and T2. No masked azotaemic cats were azotaemic at T1 but not at T2. In the azotaemic group, three cats were re-evaluated at T1: one cat had a serum creatinine concentration of 148 µmol/l, whereas the other two remained azotaemic at T1. All four cats were azotaemic at T2.

When the masked azotaemic and azotaemic groups were combined to form the hyperthyroid-CKD group (representing cats with ‘masked’ and overt CKD at baseline), there was a tendency towards higher baseline serum creatinine concentrations in the hyperthyroid-CKD group than the hyperthyroid non-azotaemic group (119 µmol/l [range 74–216] vs 97 µmol/l [range 62–118]; *P* = 0.058) (Table 1). There was no significant difference in baseline serum SDMA concentrations between hyperthyroid-CKD and hyperthyroid non-azotaemic cats (12 µg/dl [range 7–21] vs 13 µg/dl [range 11–19], respectively; *P* = 0.42) (Table 1). Other clinicopathological comparisons between the hyperthyroid-CKD and hyperthyroid non-azotaemic groups are shown in Table 1.

**Table 1:** Selected baseline biochemical parameters in 19 hyperthyroid cats classified by renal function into non-azotaemic (n = 6), masked azotaemic (n = 9) and azotaemic (n = 4) groups

Variable	Hyperthyroid non-azotaemic	Hyperthyroid masked azotaemic	Hyperthyroid azotaemic	Sig*	Hyperthyroid-CKD	Sig^†^
Age (years)	15 (14–19)	12 (9–18)	14 (8–15)	0.30	12 (8–18)	0.12
TT4 (nmol/l)	82 (49–90)	80 (49–206)^‡^	53 (47–57)^‡^	0.045	73 (47–206)	0.83
SDMA (μg/dl)	13 (11–19)	10 (7–14)^§^	16 (11–21)^§^	0.049	12 (7–21)	0.42
Creatinine (μmol/l)	97 (62–118)^¶^	99 (74–140)^∞^	169 (157–216)^¶ ∞^	0.007	119 (74–216)	0.058
Urea (mmol/l)	10.7 (6.5)	11.5 (7–15.4)	12.8 (10.3–18.3)	0.39	11.5 (7–18.3)	0.37

Data are median (range). Hyperthyroid-chronic kidney disease (CKD) represents the combined masked azotaemic and azotaemic hyperthyroid population (n = 13)

* Comparison of hyperthyroid non-azotaemic, hyperthyroid masked azotaemic and hyperthyroid azotaemic groups using the Kruskal–Wallis test

† Comparison of hyperthyroid-CKD to hyperthyroid non-azotaemic group using the Mann–Whitney U-test

‡ Significant difference in total thyroxine (TT4) between masked azotaemic and azotaemic hyperthyroid cats

§ Significant difference in symmetric dimethylarginine (SDMA) between masked azotaemic and azotaemic hyperthyroid cats

¶ Significant difference in serum creatinine between non-azotaemic and azotaemic hyperthyroid cats

∞ Significant difference in serum creatinine between masked azotaemic and azotaemic hyperthyroid cats

Sig = significance

Significant differences in baseline serum concentrations of creatinine (*P* = 0.007), SDMA (*P* = 0.049) and TT4 (*P* = 0.045) were evident between the azotaemic, masked azotaemic and non-azotaemic groups, whereas there were no differences in age (*P* = 0.30) or serum urea concentrations (*P* = 0.39) between the groups (Table 1). Serum creatinine concentrations were significantly higher in the azotaemic group than the non-azotaemic (*P* = 0.011) and the masked azotaemic groups (*P* = 0.005); however, there was no difference in serum creatinine concentrations between the masked azotaemic and non-azotaemic groups (*P* = 0.24) (Table 1). Serum SDMA concentrations were significantly higher in the azotaemic group than the masked azotaemic group (*P* = 0.03) but were not different between the azotaemic and non-azotaemic groups (*P* = 0.33), nor between the non-azotaemic and masked azotaemic groups (*P* = 0.094) (Table 1). At baseline, 3/4 cats in the azotaemic group had serum SDMA concentrations above the upper limit of the laboratory RI (14 µg/dl), whereas no masked azotaemic cats had serum SDMA concentrations above the laboratory RI. One non-azotaemic cat had serum SDMA concentration above 14 µg/dl at baseline. Azotaemic cats had significantly lower serum TT4 concentrations than masked azotaemic cats (*P* = 0.02); however, there was no difference in serum TT4 concentrations between the azotaemic and non-azotaemic groups (*P* = 0.056), nor between the non-azotaemic and masked azotaemic groups (*P* = 0.443).

When all hyperthyroid cats were combined, there was a strong inverse correlation between serum TT4 and creatinine concentrations at baseline (*r* = −0.73; *P* <0.001); however, there was no significant correlation between serum TT4 and SDMA concentrations (*r* = −0.42; *P* = 0.074). At baseline, there was a moderate positive correlation between serum SDMA and creatinine concentrations (*r* = 0.52; *P* = 0.022).

### Post-treatment changes

**Figure 1:**
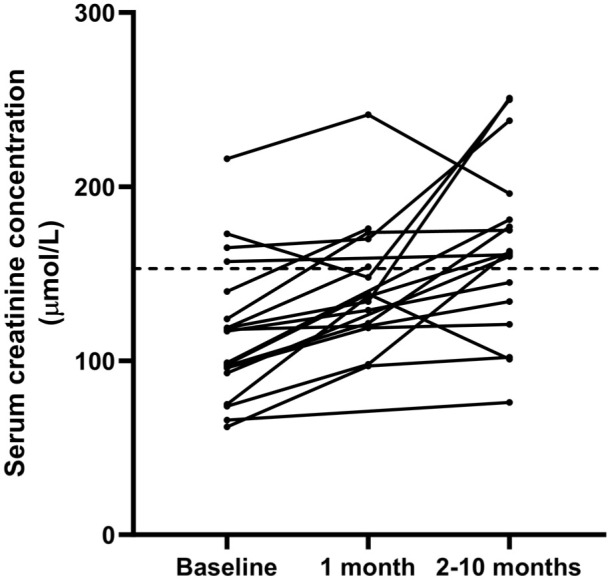
Line graph showing the change in serum creatinine concentrations measured at T0 (before treatment), T1 (~1 month after treatment) and T2 (2–9 months after treatment). Dashed black horizontal line represents the upper limit of the reference interval (153 μmol/l)

Follow-up biochemical data were available at T1 for 16 cats and at T2 for 17 cats. T2 was 86 days (range 26–282) after T1 for cats where data from both time points were available. Only two cats had USG recorded at follow-up. Serum TT4 concentrations decreased between baseline and T1 (n = 16; *P* <0.001) (Table 2) and between baseline and T2 (n = 17; *P* <0.001) (Table 2), but not between T1 and T2 (*P* = 0.753). When data from all hyperthyroid cats were combined, serum creatinine concentrations increased significantly between baseline and T1 (n = 15; *P* = 0.003) (Figure 1), and between baseline and T2 (n = 17; *P* <0.001) (Table 2, Figure 1). Serum creatinine concentrations also increased between T1 and T2 (n = 13; *P* = 0.033).

**Table 2:** Selected biochemical parameters for all hyperthyroid cats at T0 (baseline, n = 19), T1 (>1 month after treatment, n = 16) and T2 (2–9 months after treatment, n = 17)

Variable	T0	T1	T2
Days after T0	–	32 (21–47)	110 (54–303)
TT4 (nmol/l)	76 (47–206)	20 (7–40)	20 (9–38)
SDMA (μg/dl)	12 (7–21)	11 (8–29)	13 (9–24)
Creatinine (μmol/l)	117 (62–216)	137 (97–241)	162 (76–251)
Urea (mmol/l)	11.5 (6.5–18.3)	12.2 (9.9–19.5)	13.7 (6.8–22.9)
SDMA storage time (years)	–	2.9 (0.6–9.5)	1.9 (0.1–9.2)

Data are median (range)

SDMA = symmetric dimethylarginine; TT4 = total thyroxine

**Figure 2:**
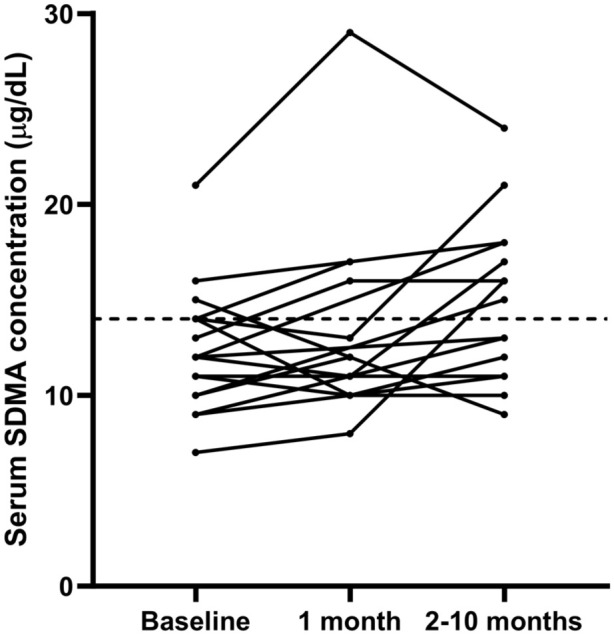
Line graph showing the change in serum symmetric dimethylarginine (SDMA) concentrations (µg/dl) measured at T0 (before treatment, n = 19), T1 (~1 month after treatment) and T2 (2–9 months after treatment). Dashed black horizontal line represents the upper limit of the reference interval (14 µg/dl)

At T1, one non-azotaemic, one masked azotaemic and one azotaemic cat had serum SDMA concentrations above the laboratory RI. At T2, 2/6 non-azotaemic cats, 3/7 masked azotaemic cats (with available data) and 3/4 azotaemic cats had serum SDMA concentrations above the laboratory RI. Serum SDMA concentrations did not change between baseline and T1 (n = 14; *P* = 0.548) (Figure 2) but increased significantly between baseline and T2 (n = 17; *P* = 0.039) (Figure 2). Serum SDMA concentrations did not change significantly between T1 and T2 (n = 12; *P* = 0.084).

## Discussion

The results of this small retrospective study indicate that both serum creatinine and SDMA concentrations increased after 2–9 months of treatment of hyperthyroidism using antithyroid medications. There was no correlation between pretreatment serum SDMA and TT4 concentrations, whereas serum creatinine concentrations showed a strong negative correlation with serum TT4 concentrations before treatment. Furthermore, neither serum creatinine nor SDMA concentrations were significantly higher in hyperthyroid cats that had concurrent CKD (overt and/or masked) compared with hyperthyroid cats that remained non-azotaemic after treatment.

It was hypothesised that both serum creatinine and SDMA concentrations would increase after restoration of euthyroidism since both correlate with GFR in healthy cats,^15,16^ and GFR would decrease after hyperthyroidism treatment.^8,9,11^ Unexpectedly, serum SDMA concentrations did not increase after approximately 1 month of treatment, whereas serum creatinine concentrations did increase over this time period. Our findings are supported by the results from some studies of radioiodine-treated hyperthyroid cats which reported that serum creatinine, but not SDMA concentrations, increased in the first month after radioiodine treatment despite a significant decrease in GFR,^21,22^ but conflict with the findings of other studies.^24^ As SDMA concentrations did increase after 2–9 months of treatment in the present study, it could be proposed that SDMA may show a more delayed response to changes in GFR than serum creatinine. However, changes in GFR and SDMA have previously been documented to stabilise 1 month after radioiodine treatment, while changes in serum creatinine associated with increased muscle mass continue for several months.^9,12,24^

Serum TT4 concentrations were inversely correlated with serum creatinine concentrations but not serum SDMA concentrations in our population. Serum TT4 concentrations are inversely correlated with GFR and serum creatinine concentrations in humans,^27^ and GFR is also documented to decrease after treatment of hyperthyroidism in cats.^9,11,28^ Therefore, we would expect an inverse correlation between serum TT4 and SDMA concentrations in hyperthyroid cats. Our findings, in conjunction with those of others, support the premise that serum SDMA concentrations are influenced by factors other than GFR in hyperthyroidism.^20–22^ In humans, circulating SDMA concentrations are increased in hyperthyroid patients, and it has been proposed that thyrotoxicosis may upregulate SDMA production.^29,30^ Decreases in serum SDMA have been reported after radioiodine treatment,^21^ and cats that have remained hyperthyroid after bilateral thyroidectomy have a steeper relationship between serum SDMA and serum creatinine.^23^ Therefore, it could be proposed that increased SDMA production in hyperthyroidism may reduce the correlation between serum SDMA concentrations and GFR in hyperthyroid cats.

In the present study, serum SDMA concentrations in initially non-azotaemic hyperthyroid cats that developed azotaemia after treatment (masked azotaemic) or the combined group of untreated hyperthyroid cats that were azotaemic at baseline or masked azotaemic (hyperthyroid-CKD group) were not significantly greater than those in untreated hyperthyroid cats that remained non-azotaemic after treatment. These data are in contrast to those from previous studies in which serum SDMA concentrations were higher in masked azotaemic cats compared with non-azotaemic cats,^18,19^ although serum SDMA concentrations were poorly sensitive in predicting post-treatment azotaemia in these radioiodine-treated cats. Our results may differ because of the smaller sample size of the present study or could reflect the presence of factors other than GFR that affect serum SDMA concentrations in hyperthyroidism. In addition, some cats that were classified as non-azotaemic had elevated serum SDMA concentrations both before and after treatment, which could reflect the presence of concurrent CKD in some cats with low muscle mass (and hence lower serum creatinine concentrations). Furthermore, some cats in the masked azotaemic and azotaemic groups had serum SDMA concentrations within the laboratory RI despite the presence of elevated serum creatinine concentrations. These findings mirror those reported in cats with azotaemic acute kidney injury,^31^ and might reflect high dispersion (the range of possible results that are feasible for a given sample based on the combination of biological and analytical variation) of serum SDMA concentrations in cats.^32^ However, correlation between serum SDMA concentrations and direct measurements of GFR would be required to confirm this hypothesis.

In the present study, a relatively high proportion (60%) of initially non-azotaemic hyperthyroid cats became azotaemic after treatment. Approximately 15–51% of initially non-azotaemic cats will become azotaemic after radioiodine, surgical or medical treatment;^6,9,19,33,34^ however, the high proportion of cats becoming azotaemic after treatment in the present study may reflect bias in the population of cats for which a full biochemistry profile was requested, rather than TT4 alone, after treatment, or could reflect the fact that cats were classified as azotaemic based on documentation of elevated serum creatinine concentrations and without documentation of USG to exclude prerenal causes of azotaemia, such as dehydration. Only 26% of cats in this study had USG measured before treatment and very few at follow-up. Hence, it is possible that some cats classified as masked azotaemic may have had prerenal azotaemia at follow-up – for example, secondary to dehydration or other reversible factors rather than a true renal azotaemia. In addition, the classification of cats according to renal status was based on serum creatinine concentrations; therefore, it is possible that some non-azotaemic cats might have had concurrent CKD. Direct measurement of GFR would be required to fully classify cats based on renal status; however, this was not possible because of the retrospective nature of the study.

This study had a number of limitations in addition to those already discussed. The three-way subdivision of the population resulted in small group sizes and, as a result, some near-significant results (such as TT4 and SDMA at baseline) may represent real biological differences that could not be demonstrated owing to the study being statistically underpowered. For example, based on the preliminary data in this study, five cats would be needed in each group for the study to be 80% powered to detect an effect size of 6 µg/dl at the 5% significance level, and 21 cats would be needed per group to detect an effect size of 3 µg/dl using the same criteria. SDMA was measured on serum samples that were frozen at –80°C for up to 10 years, and although the stability of SDMA been demonstrated for up to 24 months,^35^ the effect of longer term storage on serum SDMA concentrations is unknown; therefore, an impact of prolonged sample storage on the measured SDMA concentrations cannot be excluded. Further studies to establish the long-term stability of serum SDMA concentrations when stored frozen would be useful to help clarify the effect of prolonged sample storage on serum SDMA concentrations. In addition, serum TT4 concentrations alone were used to define thyroid status, and although all cats had serum TT4 concentrations within the RI at all time points, given the lack of concurrent serum thyroid-stimulating hormone measurements and fact that some cats with iatrogenic hypothyroidism can have low–normal serum TT4 concentrations,^36^ the presence of iatrogenic subclinical hypothyroidism, which might have reduced GFR, could not be fully excluded. Conversely, in the absence of supportive clinical parameters at follow-up, the presence of poorly controlled hyperthyroidism in cats with a TT4 towards the upper end of the RI cannot be excluded, although the RI utilised in this study was derived using a population of non-hyperthyroid and otherwise healthy senior cats.^25^ As a retrospective study, the decisions on medication, starting doses, dosing intervals and any dose adjustments were made at the discretion of the attending veterinarian and were not standardised. Additional comparisons between once- and twice-daily administration of medication would have produced small subgroups with inadequate statistical power. Finally, in the absence of additional investigations, including abdominal ultrasonography, the presence of other diseases that could have increased serum SDMA concentrations, such as lymphoma, could not be excluded.^37^

## Conclusions

Serum SDMA concentrations were not higher in hyperthyroid cats with concurrent CKD than hyperthyroid cats that remained non-azotaemic after restoration of euthyroidism, and were not useful predictors of post-treatment azotaemia in initially non-azotaemic hyperthyroid cats. Furthermore, serum SDMA concentrations did not increase in parallel with serum creatinine concentrations after 1 month of treatment with antithyroid medications and were not inversely correlated with TT4 concentrations, which could suggest that factors other than GFR influence serum SDMA concentrations in hyperthyroidism. Larger scale studies measuring serum concentrations of creatinine and SDMA in conjunction with direct measurements of GFR in methimazole/carbimazole-treated hyperthyroid cats are required to further characterise the relationship between GFR and serum SDMA concentrations in this population of hyperthyroid cats.

## Supplemental Material

S1 Raw data.
